# Microscopic Investigation of Coupled Mobilization and Blending Behaviors Between Virgin and Reclaimed Aged Asphalt Mastic

**DOI:** 10.3390/ma18163739

**Published:** 2025-08-10

**Authors:** Jiaying Zhang, Xin Qiu, Qinghong Fu, Zheyu Shen, Xuanqi Huang, Haoran Chen

**Affiliations:** Road and Traffic Engineering Institute, College of Engineering, Zhejiang Normal University, Jinhua 321004, China; zjy20040211@zjnu.edu.cn (J.Z.); shandafqh@163.com (Q.F.); szyszy@zjnu.edu.cn (Z.S.); foreternity@zjnu.edu.cn (X.H.); rtstchr@zjnu.edu.cn (H.C.)

**Keywords:** aged asphalt mastic, mobilization rate, blending degree, microscopic mechanism, correlation analysis

## Abstract

To meet the demand for sustainable pavement infrastructure, reclaimed asphalt pavement (RAP) has become a key strategy to enhance material circularity. This study investigates the coupled mobilization and blending behaviors between virgin and aged asphalt mastic in RAP systems. Fourier-Transform Infrared Spectroscopy (FTIR) was utilized to quantify the mobilization rate (MR) of aged mastic on RAP aggregate surfaces using the Composite Aging Index (CAI). Scanning Electron Microscopy (SEM) and Fluorescence Microscopy (FM), combined with digital image analysis, were employed to assess the blending interface and quantify the degree of blending (DoB). A 3D model was developed to describe the nonlinear relationship between MR and DoB. The results show that regeneration is dominated by physical diffusion, while mixing temperature has a stronger effect on MR than time. The binder interface displays a smooth transition, whereas the mastic interface exhibits a gear-like structure. DoB in the binder system is higher than that in the mastic system under the same condition, with early-stage temperature elevation playing a key role. Even near 100%, MR does not lead to full blending due to interfacial saturation. These insights are valuable for guiding the design of RAP and optimizing mixing conditions to enhance recycling efficiency in practical applications.

## 1. Introduction

In recent years, the efficient utilization of reclaimed asphalt pavement (RAP) materials has attracted growing international attention as a critical strategy for promoting sustainability in pavement engineering. Countries and regions such as the United States, Europe and Australia have recognized the environmental and economic benefits of high-percentage RAP incorporation, making it a prominent topic in pavement material research and infrastructure practice [1,2,3]. Although the extent of large-scale application varies across regions, the pursuit of high-value recycling and long-term performance improvement of RAP-based mixtures remains a shared global concern, especially under the broader goals of reducing carbon emissions and conserving natural resources.

Recently, plant-mixed hot regeneration technology has achieved significant progress in asphalt pavement applications due to its advantages of high RAP utilization rate, superior quality control of reclaimed asphalt mixtures, and strong compatibility [4,5,6]. This approach not only contributes to reducing the consumption of virgin resources but also aligns with circular economy principles, thereby decreasing the environmental burden associated with raw material extraction and waste disposal. However, in current hot recycling processes, mechanical mixing and high temperatures activate and detach only part of the aged binder from RAP aggregates, enabling limited diffusion and blending with the virgin binder. This results in a heterogeneous multiphase composite asphalt system within reclaimed asphalt mixtures, consisting of aged asphalt binder, virgin asphalt binder, and partially blended regenerated asphalt binder. This heterogeneity tends to form weak interfacial and interlayer zones with aggregates, which are highly susceptible to premature functional failure in reclaimed asphalt mixtures [7,8,9]. Furthermore, in asphalt mixture dispersion systems, the actual binder responsible for bonding aggregates, filling voids, and transferring loads is mastic rather than pure binder. Due to the presence of highly viscous aged mastic on RAP surfaces, blending with virgin mastic becomes even more difficult, further aggravating interfacial incompatibility and weak zone formation [10,11,12]. Therefore, targeted research on aged asphalt mastic mobilization and blending is critical for enhancing reclaimed asphalt mixture performance while advancing sustainable material reuse and reducing the environmental footprint of asphalt pavements.

Studies have shown that incorporating RAP into virgin asphalt binder involves a two-stage mechanism: firstly, the activation and migration of aged asphalt mastic from RAP surfaces; secondly, the interdiffusion and blending between virgin-aged asphalt mastic [13]. During the activation stage, the mobilization rate (MR, indicating the proportion of aged binder transferred to virgin binder) serves as a key parameter for determining the appropriate amount of virgin binder in reclaimed asphalt mixtures [14]. In the process of mixing, the regenerated asphalt binder (RAb) is activated and separated from RAP surfaces, transferred to the surfaces of virgin aggregates, and subsequently undergoes diffusion and blending with virgin asphalt binder [15,16]. During the blending phase, the activated RAb interpenetrates and diffuses with virgin asphalt binder, gradually forming a homogenized blended asphalt mastic. The extent to which virgin and aged asphalt mastics integrate into a regenerated asphalt mastic is defined as the degree of blending (DoB, reflecting how uniformly the virgin and aged binders are blended) [17,18]. Studies demonstrate that the DoB is a key factor influencing the pavement performance of reclaimed asphalt mixtures [19,20]. A high content of aged asphalt mastic or a low activation level of RAb can lead to a reduction in the DoB [21,22], thereby compromising the quality of the reclaimed asphalt mixture. It is evident that the activation and blending behaviors of RAP materials are closely interrelated; the MR determines the required virgin asphalt binder dosage in regenerated mixtures, while the DoB determines the performance efficacy of the regenerated mixtures [23,24,25].

In recent years, substantial efforts have been made to investigate the activation and blending mechanisms of RAP materials, leading to notable advancements in this field. Current methodologies primarily include mechanical approaches [24,26,27], chemical approaches [23,28,29], visualization approaches [26,30,31], and mechanistic approaches [32,33,34]. Among these, microscopic characterization methods offer intuitive and quantitative insights into activation and blending behaviors, enabling the assessment of diffusion depth, degree of blending, and spatial homogeneity between virgin and aged asphalt binder or mastic at the microscale. Many researchers have utilized microscopic techniques, such as Fourier-Transform Infrared Spectroscopy (FTIR), Scanning Electron Microscopy (SEM), and Fluorescence Microscopy (FM), to characterize the diffusion and interfacial integration between virgin aged binder and mastic components. These studies provide more accurate and comprehensive interpretations of the blending and regeneration behaviors between the mixtures, advancing the understanding of RAP regenerating mechanisms.

In terms of the activation and diffusion degree (reflecting how effectively aged binder is reactivated and intermixed with virgin binder), FTIR has been widely applied due to its capability to accurately characterize the microstructure composition, functional group characteristics of materials, and the physical–chemical interaction mechanisms in the blending process of multiphase materials [35,36,37,38]. Li et al. [39] utilized FTIR to characterize the influence of the activation degree of waste crumb rubber-modified asphalt binder on the functional groups, cross-linking, and viscoelasticity of crumb rubber. Sreeram et al. [40] applied ATR-FTIR to assess the activation rate of a RAP binder from different sources, showing that activation is temperature-dependent and can be enhanced by warm-mix additives. Wu et al. [41] utilized FTIR to demonstrate that microwave induction technology effectively improves the activation rate of RAP binder. Lei et al. [42] applied FTIR analysis to investigate the transfer behavior and activation degree differences of aged binder, revealing that the transfer of aged binder in reclaimed hot-mix asphalt mixtures significantly influences the skeletal structure. Li et al. [43] identified a direct relationship between the carbonyl index and aging degree, reporting that increased mixing temperatures diminish the carbonyl index while promoting the activation of asphalt binder. Collectively, these studies confirm that infrared spectroscopy enables precise measurement of migration depth and functional group content in regenerated asphalt mastic, offering quantitative insight into aged binder mobilization as well as the diffusion and permeation behavior between virgin and aged asphalt mastic.

Building upon the chemical-level observations provided by FTIR, researchers have also focused on the physical structure of the blending interface. In terms of interfacial fusion states (degree of physical continuity and structural integration at the binder interface), researchers have utilized FM and SEM to study the morphological characteristics of the blending interface between virgin aged asphalt binder or mastic, elucidating the migration mechanisms of virgin binder. Ding et al. [15,44,45] extracted the mean gray value (MGV) from fluorescence images to evaluate the DoB between virgin and aged binders. The results suggest that extending the mixing duration and elevating the processing temperature can significantly enhance the mobilization efficiency of aged binder within RAP materials. Zou et al. [46] achieved 3D visualization and quantitative assessment of virgin aged binder interaction at the asphalt–aggregate interface by applying LSCM integrated with fluorescent particle techniques. Tao et al. [47] introduced a quantitative method using grayscale analysis of fluorescence microscopy images to quantify the blending effectiveness of virgin aged binder systems. SEM is often combined with Energy-Dispersive Spectroscopy (EDS) to examine the interfacial transition region between virgin aged binders. Jiang et al. [48] employed SEM–EDS techniques to show that the DoB between binders decreases sharply with higher RAP content, along with a decline in uniform binder distribution. Abdalfattah et al. [49] utilized Energy-Dispersive X-ray Spectroscopy (EDX) to quantify the DoB between virgin and RAP binders. It was indicated that a higher content of RAP and elevated Performance Grade (PG) of the RAP binder can lead to decreased average blending efficiency. Jiang et al. [48] employed titanium dioxide (TiO_2_) as the tracing agent [26], adopting the titanium-to-sulfur (Ti/S) mass ratio as a quantitative marker for blending assessment. SEM/EDS analysis revealed a significant decrease in the DoB between virgin aged binder as RAP content rises, accompanied by a decline in the even distribution of the blended binder. Based on these studies, microscopic testing techniques, as rapid and accessible tools, are widely used to characterize the distribution state, penetration depth, and other features of the migration process between aged asphalt binder and mastic on RAP surfaces and virgin asphalt binder. These techniques provide distinct benefits in elucidating the blending mechanisms between virgin aged binder and mastic.

Undoubtedly, researchers have carried out numerous studies addressing activation or blending separately, achieving significant advancements. However, in reality, the mobilization of aged asphalt mastic on RAP surfaces and its blending process are inherently interconnected during regeneration. Despite this, most existing research isolates the analysis of activation and blending, lacking a systematic and comprehensive understanding of their interactive mechanisms. Therefore, selecting and integrating effective microscopic testing and analytical methods to investigate the full process of activation and blending between virgin and aged asphalt mastic, while quantitatively evaluating their interdependencies, are not only critical for enhancing activation blending efficiency and improving the performance of reclaimed asphalt mixtures at the material level but also essential for guiding practical engineering decisions. Such insights can help optimize mix design parameters, processing conditions, and construction practices, ultimately leading to more durable, cost-effective, and sustainable pavement infrastructures that reduce reliance on virgin materials, lower environmental impacts, and promote the high-value utilization of recycled resources.

In summary, unlike previous studies that focused separately on activation or blending processes, this study conducts a coupled analysis and establishes a nonlinear correlation model by integrating FTIR, SEM, and FM techniques, thereby supporting the long-term performance and practical application of high-RAP-content pavements.

The main contents of this study are as follows:

1. To investigate the mobilization behavior of aged asphalt mastic on RAP aggregate surfaces under varying mixing conditions, through analysis of chemical functional group evolution using FTIR spectroscopy.
2. To characterize the interfacial diffusion and migration processes between virgin and aged asphalt mastic by examining the micro-morphological features of their blended interfaces via SEM.
3. To quantify the DoB between virgin and aged asphalt mastic based on fluorescence characteristics, using image-based analysis techniques integrated with FM were performed using OPLENIC Echo-Imager 2.18 software and HALCON machine vision 12.0 software.
4. To establish a coupling model that captures the nonlinear effects of mixing conditions on activation and blending performance, aiming to clarify the underlying interaction mechanisms between these two processes.

## 2. Materials and Methods

### 2.1. Materials

#### 2.1.1. RAP

The RAP material was sourced from surface layers of an expressway in Jinhua, Zhejiang Province. Prior to testing, the RAP underwent extraction and distillation processes. Specifically, the RAP was soaked in trichloroethylene solvent to dissolve the aged asphalt binder on its surface, followed by an extraction and recovery procedure to collect the aged asphalt binder. This method allows direct observation of the environmental impacts on the pavement material during service life. Zhao et al. [50] confirmed that trichloroethylene is considered the best solvent for asphalt extraction, ensuring complete dissolution without preferential decomposition.

#### 2.1.2. Asphalt Binder

The virgin binder employed in this study was Zhenhai 70#, sourced from China. The composition is chiefly hydrocarbons varying in molecular weight, together with non-metallic derivatives, resulting in a complex chemical composition and microstructure. These inherent properties influence its compatibility and diffusion behavior when blended with aged asphalt mastic present on RAP, thereby influencing the overall behavior of the reclaimed asphalt mixture. The aged binder was directly recovered from RAP through solvent extraction, rather than artificially aged in the laboratory. During its service life, asphalt binder undergoes irreversible aging due to prolonged exposure to environmental influences and mechanical loading, which causes notable declines in mechanical properties and durability. A set of standard experiments was conducted to quantitatively assess the performance differences between virgin aged binders. The corresponding technical indicators are presented in Table 1.

#### 2.1.3. Aged Asphalt Mastic

The aged asphalt mastic was formulated using aged asphalt binder obtained through extraction and distillation [55] and limestone filler at a binder-to-filler ratio of 1.2 [56]. The preparation procedure was as follows: The limestone filler was passed through a 0.075 mm standard mesh, subsequently dried at 150 °C in oven for 2 h to remove moisture. The aged binder was warmed at 135 °C in oven for 2 h to ensure optimal fluidity. During preparation, the pre-heated filler was gradually added in small increments to the rejuvenated asphalt under continuous mechanical shearing. The mixture was sheared at 1000 rpm for 30 min until no visible bubbles were observed and this step could prevent performance discrepancies caused by uneven filler distribution [56]. The prepared mastic should be immediately transferred to a sealed aluminum container and stored at room temperature for subsequent testing.

#### 2.1.4. Regenerated Asphalt Mastic

To measure the activation degree of aged asphalt mastic on RAP surface under actual mixing conditions, the following mixing process to prepare regenerated asphalt mastic (blended asphalt mastic) was conducted. And to facilitate clear distinction between RAP and virgin aggregates after mixing, their particle sizes were controlled to be significantly different. In this mixing process, 3–5 mm RAP and 10–19 mm aggregates were selected. Their characteristics are shown in Table 2.

**Figure 1:**
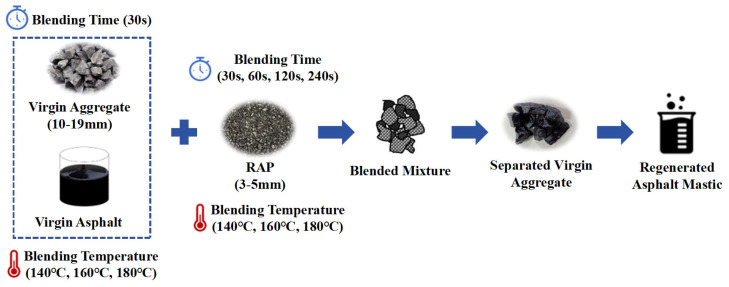
Preparation of regenerated asphalt mastic.

During the initial stage of sample preparation, the mixing time was set to 30 s, 60 s, 120 s, and 240 s, while the mixing temperature was maintained at 140 °C, 160 °C and 180 °C. Before preparing the samples, the virgin aggregate and RAP were separately heated in an oven at temperatures of 200 °C and 120 °C and held at these conditions for two hours. Based on the most effective mixing sequence identified in previous studies [57], the virgin aggregate was initially mixed with virgin asphalt binder for 30 s at 140 °C, 160 °C or 180 °C until homogenized, after which RAP was introduced into the mixture, as illustrated in Figure 1. This approach ensures a uniform coating of virgin binder over the aggregates, improves the adhesion of fine RAP particles to the virgin surfaces, and extends the effective mixing time for aged mastic migration. Consequently, this mixing sequence was adopted to explore the effect of mixing duration (0–240 s) and temperature on transfer efficiency. For each mixing condition, three replicate samples were analyzed, and the average values were calculated to guarantee the reliability and consistency of the results.

After the specified mixing time and temperature, the coarse and fine aggregates in the reclaimed asphalt mixture were manually separated. The coarse aggregates were processed using a fully automatic asphalt binder analyzer to isolate aggregates, mineral fillers, and asphalt binder, yielding a mixed solution containing trichloroethylene. Following ASTM D7906-14 [58], the regenerated asphalt binder was extracted using rotary evaporation. The mineral filler was incorporated, leading to the development of regenerated asphalt mastic characterized by a filler-to-binder ratio of 1.2. During mixing, the regenerated asphalt mastic formed on the surfaces of virgin aggregates is composed of both migrated aged mastic and virgin binder. Extracting the blended asphalt mastic from RAP would include non-transferred regenerated asphalt mastic; thus, the blended asphalt mastic on virgin aggregate surfaces was specifically targeted for separation.

### 2.2. Experimental Methods

#### 2.2.1. FTIR

In the study of reclaimed asphalt mixtures, the activation of RAP asphalt binder plays a vital role in regeneration effectiveness. This step acts as the foundation for the mixing behavior between virgin aged asphalt mastic during regeneration. The content of mobilized binder directly determines the blending efficiency between virgin aged asphalt mastic, thus impacting the road performance of reclaimed asphalt mixtures. The mobilization rate (MR) is defined as the proportion of the mobilized RAP binder mass relative to the total RAP binder mass, reflecting the extent of binder activation during the regenerating process. Zhao et al. and Ding et al. [20,22,26] derived Equation (1) for calculating the MR through a series of equation derivations:

(1) $$MR=\frac{\alpha \times P_{v}}{(1-\alpha )\times P_{r}},$$

where *α* represents the fraction of mobilized RAP binder compared to the total binder blend, while *P_v_* and *P_r_* denote fixed design values that specify the mass proportions of virgin and RAP binders in the mixture.

However, it is challenging to directly measure the quantity of aged binder that softens and delaminates from the RAP surface, making the quantitative evaluation of its mobilization degree inherently difficult. Fortunately, studies have demonstrated that the aging functional group indices in the blended asphalt are directly linked to the amount of aged binder [22,40,59,60]. This allows indirect reflection of the mobilized aged binder content through the analysis of these functional group indices in the blended asphalt binder. Typically, the carbonyl (C=O) and sulfoxide (S=O) functional groups exhibit prominent peaks in aged asphalt binder [61]. Higher RAP binder content in the aged asphalt binder corresponds to increased carbonyl and sulfoxide indices. This research utilizes the Composite Aging Index (CAI), defined as the ratio of the peak areas of C=O (1700 cm^−1^) and S=O (1030 cm^−1^) to that of C-C (1458 cm^−1^), as the quantitative indicator to analyze changes in asphalt binder during aging and regenerating. The calculation formula for the CAI, Equation (2), is expressed as follows:

(2) $$CAI=\frac{\left(A_{1700}+A_{1030}\right)}{A_{1458}},$$

where *CAI* denotes the Composite Aging Index, and *A* represents the peak areas of specific spectral bands in the sample.

**Figure 2:**
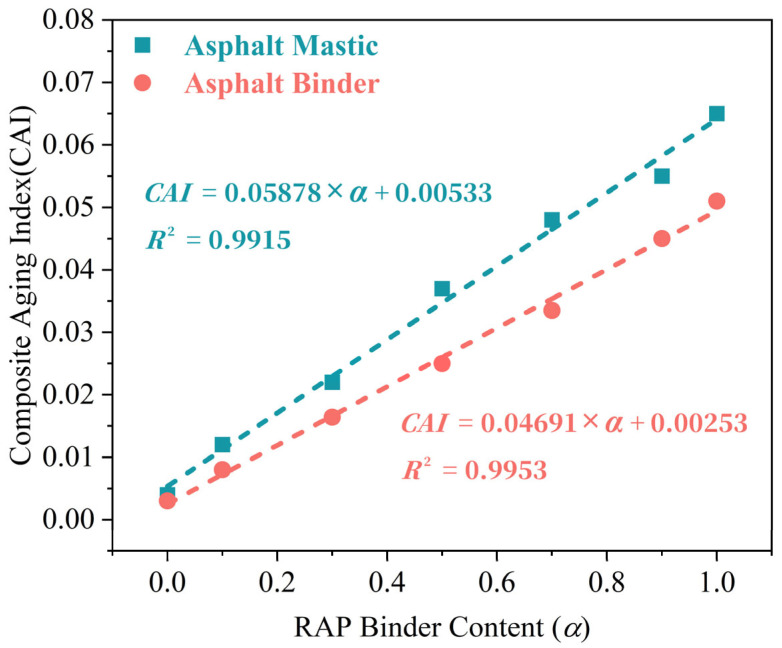
Relationship between *α* and *CAI* values of asphalt binder and mastic.

To establish the relationship between CAI values and RAP content (*α*), regenerated asphalt binder and mastic specimens were created through mixing RAP binder and mastic with virgin asphalt binder at predetermined ratios. With RAP content systematically varied from 0% to 100%, Figure 2 illustrates the CAI measurements of each regenerated asphalt binder and mastic specimens.

*α* serves as an essential factor for calculating the MR, and FTIR enables precise quantification of the CAI. The equations in Figure 2 demonstrate linear correlations between α and CAI; therefore, the CAI value can be substituted for α to establish the functional relationship MR = f(CAI), allowing for indirect determination of the MR value. Thereby, the relationships between the MR of aged binder and mastic on RAP surfaces and CAI values can be derived, as, respectively, shown in Equations (3) and (4):

(3) $$MR_{Asphalt}=\frac{P_{v}\times (CAI-0.00253)}{P_{r}\times (0.04944-CAI)},$$

(4) $$MR_{Asphalt\;Mastic}=\frac{P_{v}\times (CAI-0.00533)}{P_{r}\times (0.06411-CAI)},$$

To evaluate the activation and migration extent of aged asphalt mastic during real mixing, Fourier-Transform Infrared (FTIR) Spectroscopy (Nicolet iS5, Thermo Fisher Scientific, Waltham, MA, USA) was used. The following parameters were set through the ATR mode of the Fourier Infrared Spectrometer: Attenuated Total Reflection (ATR) analysis was conducted using an ATR-Ge crystal, acquiring spectra across the 4000–600 cm^−1^ spectral range at 8 cm^−1^ resolution with 0.964 cm^−1^ data spacing through 16 co-added scans. For mineral filler testing in transmission mode, the filler was mixed with potassium bromide (KBr) particles at a 1:100 ratio, thoroughly ground and homogenized in an agate mastic to ensure uniform dispersion of mineral filler particles within KBr. The mixture was then compressed into a circular translucent pellet using a hydraulic press. The OMNIIC 9.0 software was employed to calculate the peak areas of C=O, S=O and C-C bands, thereby determining the CAI value of the regenerated asphalt mastic extracted from virgin aggregates and evaluating the corresponding MR results.

Although FTIR-CAI provides an indirect estimation of the MR based on the relative intensities of oxidation-related functional groups (C=O and S=O), its reliability has been supported in previous studies through comparisons with staged extraction and tracer-based techniques. In this study, calibration was performed by preparing a series of asphalt binder and mastic blends with known RAP contents (0–100%) to establish the CAI–RAP content relationship (Figure 2), confirming the method’s sensitivity and applicability. While gravimetric and tracer-based methods (e.g., EDS elemental ratios such as Ti/S or Ca/S) can offer more direct quantification of mobilization, they are often limited by binder heterogeneity or the need to introduce exogenous elements that may affect material behavior. Considering these limitations, the FTIR-CAI method was selected in this work due to its non-destructive nature, high resolution, and validated performance. Future research may further enhance the robustness of MR quantification through cross-validation with tracer-assisted techniques.

#### 2.2.2. SEM

To reveal the boundary between virgin aged binder or mastic, and the penetration behavior of virgin binder into aged binder or mastic, the Scanning Electron Microscopy (SEM) apparatus (COXEM EM30-AX Plus, COXEM Co., Ltd., Daejeon, Korea) was used to investigate the microstructural morphology and the DoB of virgin aged binder or mastic under various blending conditions.

**Figure 3:**
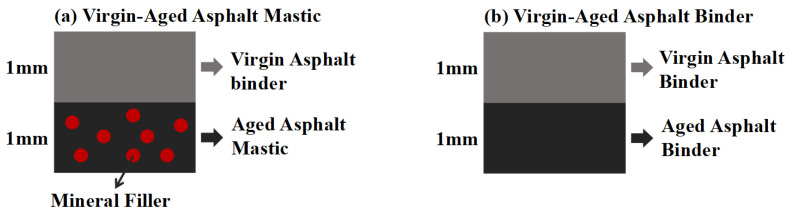
Preparation of SEM specimens.

The SEM sample is shown in Figure 3, and the preparation procedure for the samples is detailed as follows: (1) virgin and aged asphalt binder or aged asphalt mastic were molded into 1 mm thick samples using custom molds and cooled at −10 °C for ten minutes; (2) the two layers were stacked and heated at 140 °C, 160 °C or 180 °C for 30 s, 60 s, 120 s or 240 s to achieve varying blending degrees; (3) after cooling at −10 °C for 10 min, specimens were fractured at the center, and the cross-sections were treated with liquid nitrogen to obtain smooth surfaces; (4) fixed the fracture surface on SEM mold to observe the interface morphology of the blended sample. Non-blended and fully blended specimens were prepared as references: The non-blended samples followed the aforementioned preparation protocol without a temperature-controlled blending process. Fully homogenized specimens were prepared by pre-heating virgin and aged binder or mastic to 160 °C, followed by high shear mixing at 3000 rpm for 10 min and then cutting into appropriate size for SEM observation.

For SEM imaging, specimens were mounted on metal stubs with conductive adhesive, sputter-coated with gold for 2 min under vacuum, and loaded into the SEM chamber. Observations were conducted at 200× and 500× magnifications under 20 kV. To minimize experimental random error and ensure result representativeness, three sets of repeated experiments were conducted. In this study, specimens during the beginning and concluding phases of blending under the condition of 180 °C were selected for observation to investigate the blending mechanisms between virgin aged binder and mastic.

Despite its effectiveness in visualizing interfacial morphology, the SEM method has inherent limitations. The cold-fracture technique, while useful for preparing smooth cross-sections, may introduce artifacts or microstructural distortions that affect interface interpretation. Additionally, SEM provides only two-dimensional surface images, which may not fully capture the three-dimensional blending behavior between virgin and aged binders. The gold sputtering and high-vacuum conditions required for imaging may also alter surface characteristics at the microscale. Therefore, SEM results should be interpreted with caution and, where possible, supported by complementary characterization techniques.

#### 2.2.3. FM

**Figure 4:**
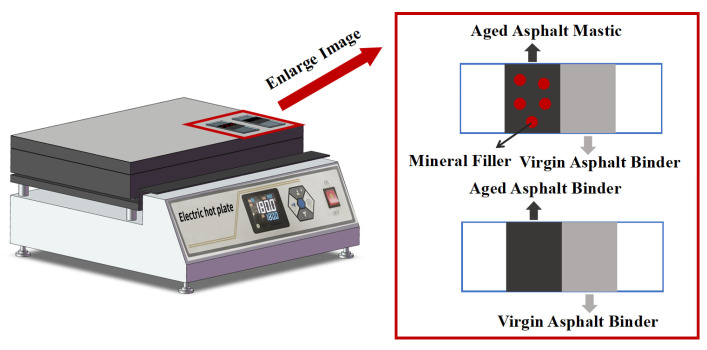
Preparation of FM samples.

Since transferred RAP binder may not fully blend with virgin asphalt binder, a fluorescence microscope (Leica DM2700P, Leica Microsystems GmbH, Wetzlar, Germany) with OPLENIC Echo-Imager 2.18 software was employed to capture microscopic images of virgin aged binder or mastic. Through this microscopic characterization, the blending behavior mechanism between these two materials was analyzed, and their blending degree was evaluated. The FM specimen preparation protocol is as follows: (1) a glass slide was horizontally positioned, and a thin blade was placed at its center; (2) aged asphalt binder or aged asphalt mastic was applied to the left side of the blade, while virgin asphalt binder was coated on the right side to simulate initial contact; (3) the blade was removed, and the slide was heated on an electric furnace under controlled blending conditions (blending time: 30 s, 60 s, 120 s, 240 s and blending temperature: 140 °C, 160 °C, 180 °C) to allow contact and blending of the two materials at the center (Figure 4).

Throughout the testing procedure, the samples were mounted on the FM rotating stage. Initially, observations were made in transmission mode using 100× eyepieces and 10× objective lenses. Then, the system was switched to fluorescence mode by activating the light source. Suitable observation areas were selected to capture morphological characteristic images of the interface between virgin aged binder and mastic. To minimize experimental random error and ensure result representativeness, three sets of repeated experiments were conducted. This approach enabled precise characterization of the microstructural features at the blending zone and the DoB between virgin aged binder and mastic.

While FM provides valuable visual insights into the blending interface, it has notable limitations. Its lower resolution compared to SEM may hinder the identification of fine interfacial features. Fluorescence intensity and contrast are also sensitive to sample thickness, binder composition, and excitation conditions. Moreover, the simplified sample preparation—placing binder side by side and heating—may not fully replicate field blending conditions. These factors can introduce uncertainties in interpreting the actual blending extent. Therefore, FM observations should be viewed with caution and preferably validated using complementary methods such as SEM or digital image analysis.

#### 2.2.4. Digital Image Technique

To further quantitatively analyze the extent of blending of virgin aged binder or mastic, the FM samples were analyzed using Halcon 12.0 machine vision software (MV tec, Germany) combined with the optical characteristic parameter (MGV) for image processing of blending interfaces. Fluorescence images of the virgin samples served as reference groups to evaluate the DoB between virgin aged asphalt binder and mastic. The test generated two optical parameters: MGV* (virgin binder) and MGV (virgin aged binder or mastic). The blending degree of virgin aged binder or mastic under FM testing was ultimately calculated using Equation (5):

(5) $$DOB=\frac{MGV}{MGV^{*}}$$

**Figure 5:**
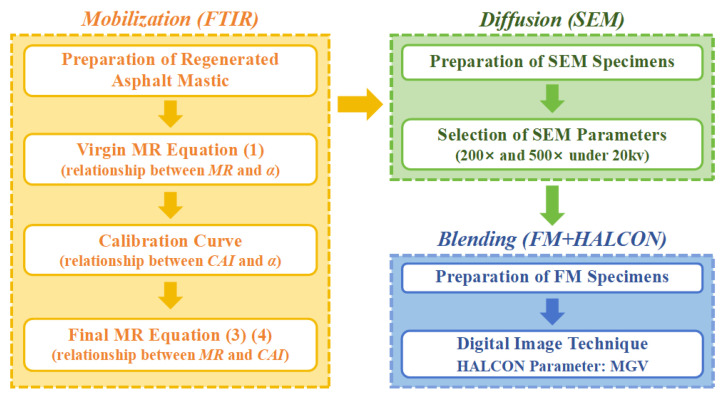
Flowchart of Section 2.

The overall workflow of the experimental program, encompassing the mobilization, diffusion, and blending analyses, is presented in Figure 5. This schematic integrates the specimen preparation procedures, instrument parameter settings, and analytical approaches employed at each stage, thereby providing a concise visual summary of the methodology framework adopted in this study.

## 3. Results and Discussion

### 3.1. Analysis of Activation and Transfer Behaviors

#### 3.1.1. Microstructure Characteristics

**Figure 6:**
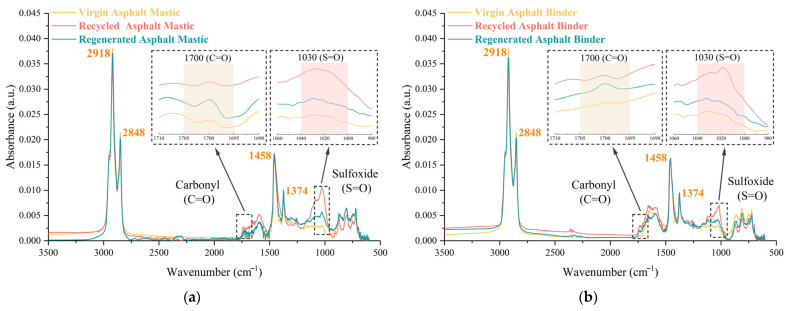
FTIR analysis: (**a**) asphalt mastic; (**b**) asphalt binder.

As illustrated in Figure 6, the absorption peaks at 2918 cm^−1^ (asymmetric -CH_2_-), 2848 cm^−1^ (symmetric -CH_3_), 1458 cm^−1^ (bending -CH_2_-), and 1374 cm^−1^ (bending -CH_3_) related to C-H bonds remain largely unchanged across virgin, aged, and regenerated asphalt samples, attributed to chemical stability. In contrast, pronounced absorption bands corresponding to carbonyl (C=O, 1700 cm^−1^) and sulfoxide (S=O, 1030 cm^−1^) are evident in both aged and regenerated samples, while their intensities in virgin asphalt mastic are substantially lower. This indicates that oxidative aging is the main cause of C=O and S=O group formation, driven by the incorporation of oxygen into molecular structures. Although the volatilization of light components and depletion of aromatics do not directly produce these functional groups, they contribute indirectly by concentrating the polar, oxygen-containing species, particularly through the enrichment and aggregation of asphaltenes [62].

As observed in Figure 6a, the regenerated asphalt mastic exhibits absorption peak intensities at the C=O and S=O regions that lie between those of the virgin and aged samples, indicating that the aged components are partially diluted during mixing with the virgin asphalt mastic. This is primarily due to limited molecular interdiffusion between the aged and virgin phases, which restricts the redistribution of polar oxygen-containing compounds. Additionally, no new absorption peaks are observed, confirming that the regeneration process is primarily driven by physical diffusion rather than chemical reactions. In Figure 6b, the regenerated binder also shows moderate intensities at the C=O and S=O bands with no new absorption peaks. Compared with Figure 6a, it is obvious that both asphalt mastic and binder systems exhibit comparable trends in terms of aging-induced peak increase and regeneration-induced attenuation at characteristic oxidative bands. However, the regenerated asphalt binder shows a more pronounced reduction in C=O and S=O intensities compared to the mastic, suggesting more effective interdiffusion and compatibility. It is attributed to the absence of mineral fillers in binder systems, allowing for better molecular mobility and contact between the virgin and aged components. In contrast, the mastic structure limits such interactions, leading to relatively weaker regeneration efficiency. These differences show the critical role of microstructural composition in influencing the regeneration behavior and mixing efficiency.

Based on the FTIR results, the following conclusions can be drawn: (1) The intensities of the carbonyl and sulfoxide bands are strongly correlated with the degree of oxidative aging, confirming their diagnostic value in chemical aging evaluation. (2) The partial recovery observed in regenerated materials is attributed to physical blending and interdiffusion, rather than a chemical reversal of oxidation. These findings validate the use of the CAI as a robust quantitative metric for evaluating the extent of aging and the effectiveness of rejuvenation. Furthermore, the continued presence of oxidation products underscores the limitations of purely physical regeneration. Without the incorporation of compatibility-enhancing additives or chemically active rejuvenators, aged components retain a memory of degradation, which may adversely affect long-term durability and resistance to further aging. Therefore, optimizing the mobilization and blending mechanisms through advanced materials and chemical rejuvenators is essential for achieving high-performance, durable, and environmentally responsible asphalt regenerating practices.

#### 3.1.2. Mobilization Rate

**Figure 7:**
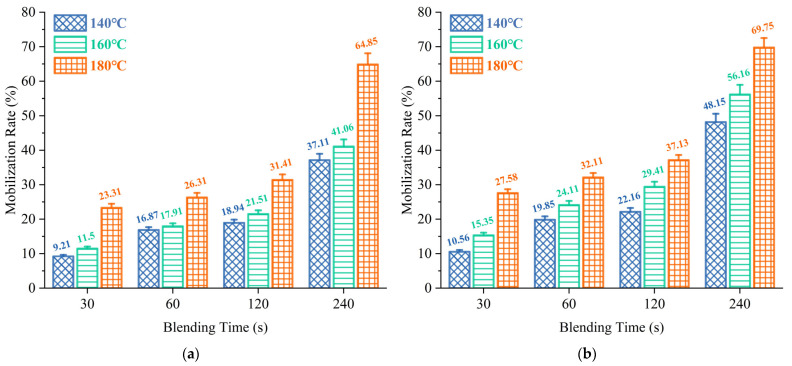
MR under different mixing conditions: (**a**) asphalt mastic; (**b**) asphalt binder.

Figure 7a indicates that heating from 140 °C to 160 °C can lead to an MR enhancement of approximately 25%, while further heating to 180 °C yields more than a 45% improvement. This is primarily attributed to thermal softening and viscosity reduction in the aged asphalt mastic at elevated temperatures, which enhances its molecular mobility and weakens its adhesion to the RAP, thereby facilitating migration toward the virgin asphalt mastic. Similarly, the MR exhibits a strong positive correlation with mixing time. In the early mixing stages (30–120 s), MR increases gradually due to insufficient interaction time and limited contact interface. However, during prolonged mixing (120–240 s), the MR surges by over 90%, resulting from the thinning of the aged mastic film on the RAP surface. This film thinning increases the interfacial area for diffusion, enabling virgin asphalt binder to more effectively infiltrate and activate the aged mastic layer, thus accelerating its detachment and transfer [63]. Figure 7b reveals that aged asphalt binder consistently shows higher MR values than aged asphalt mastic. At 180 °C and 240 s, the MR of asphalt exceeds 65%, whereas that of mastic remains significantly lower. This discrepancy arises from the absence of mineral fillers in asphalt, which reduces its viscosity and internal friction, making it more susceptible to flow and interdiffusion. In contrast, the presence of mineral filler in mastic increases its structural stiffness and coats the asphalt, forming physical barriers that hinder molecular diffusion and reduce the interfacial compatibility between virgin and aged components.

It is worth mentioning that, under all tested conditions, the MR of aged asphalt binder and mastic does not exceed 70%, indicating that a large portion of aged asphalt binder remains inactive and strongly adhered to RAP. This is mainly due to the fact that with high molecular weight, oxidized compounds exhibit poor compatibility with virgin asphalt binder, resulting in limited participation in regeneration and a low effective asphalt content in RAP. Although increasing mixing temperature and time can improve activation, the rate rarely reaches 100% in practice. Additionally, the presence of mineral fillers in asphalt mastic further hinders activation. Therefore, the actual activation degree and its interaction with fillers must be considered, and the virgin asphalt binder dosage is optimized to enhance the durability and crack resistance of regenerated mixtures.

### 3.2. Analysis of Diffusion Behaviors Based on SEM

**Figure 8:**
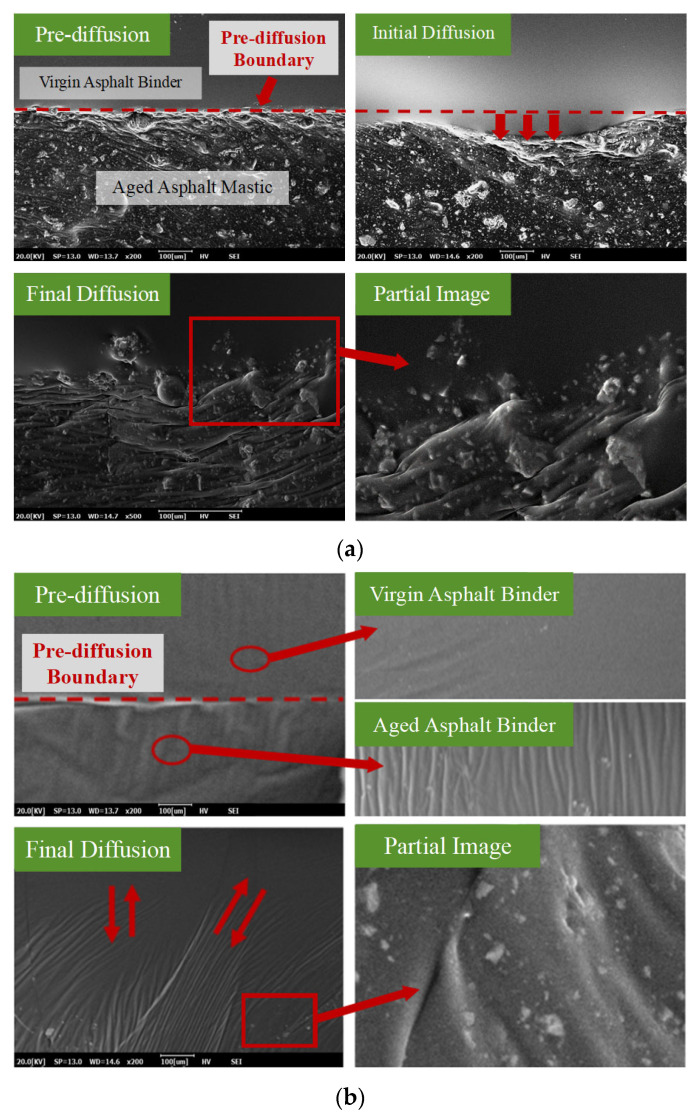
SEM images of the diffusion interface: (**a**) asphalt mastic; (**b**) asphalt binder.

Figure 8 presents the SEM images of the diffusion interfaces between virgin aged materials to investigate their diffusion and fusion behaviors. From Figure 8a, it is obvious that the virgin asphalt mastic (upper layer) and aged asphalt mastic (lower layer) are initially separated by a clear and linear boundary, indicating limited interaction at the early diffusion stage. During the initial diffusion stage, the boundary gradually shifts and appears irregularly curved, with the virgin binder showing a tendency to migrate into the aged mastic. In SEM images of the final diffusion stage, the virgin asphalt binder and aged asphalt mastic become interwoven, resulting in an indistinct boundary. The diffusion interface between the two materials resembles gear-like interlocking. Moreover, the migration depth of the aged mastic into the virgin binder is smaller than that of the virgin binder into the aged mastic. This difference is likely due to the higher proportion of macromolecules in the aged asphalt mastic, which restricts molecular mobility and leads to different migration depths. Observations near the boundary in the final blending stage reveal that the blending transition zone gradually recovers from dense wrinkles to a smoother texture. Aggregated mineral fillers are enveloped by the virgin asphalt binder, hindering the migration of the virgin asphalt binder. On the contrary, from Figure 8b, it can be observed that the migration interface between virgin aged asphalt binder exhibits a more continuous and gradual transition compared to the mastic system. No distinct boundary is present, and the interface appears smooth, indicating a higher degree of diffusion. The virgin asphalt binder tends to infiltrate deeply into the aged asphalt binder layer, forming a seamless interfacial region. This phenomenon is attributed to the superior fluidity and lower viscosity of the aged binder, which facilitates molecular diffusion and promotes a more uniform diffusion process.

By comparing Figure 8a,b, it is evident that significant differences exist in the interfacial migration behavior between virgin aged systems. The asphalt binder system demonstrates better compatibility, higher diffusion efficiency, and a more uniform interfacial structure, whereas the asphalt mastic system suffers from reduced diffusion ability due to the physical barriers formed by mineral fillers and the restricted mobility of macromolecules. In conclusion, the presence of mineral filler and the macromolecular structure in the aged asphalt mastic limit its compatibility and fusion efficiency with virgin asphalt binder, whereas aged asphalt binder has better flow characteristics, facilitating the formation of a more continuous and integrated diffusion interface.

### 3.3. Analysis of the Blending Behavior Based on FM

#### 3.3.1. Dynamic Blending Process

**Figure 9:**
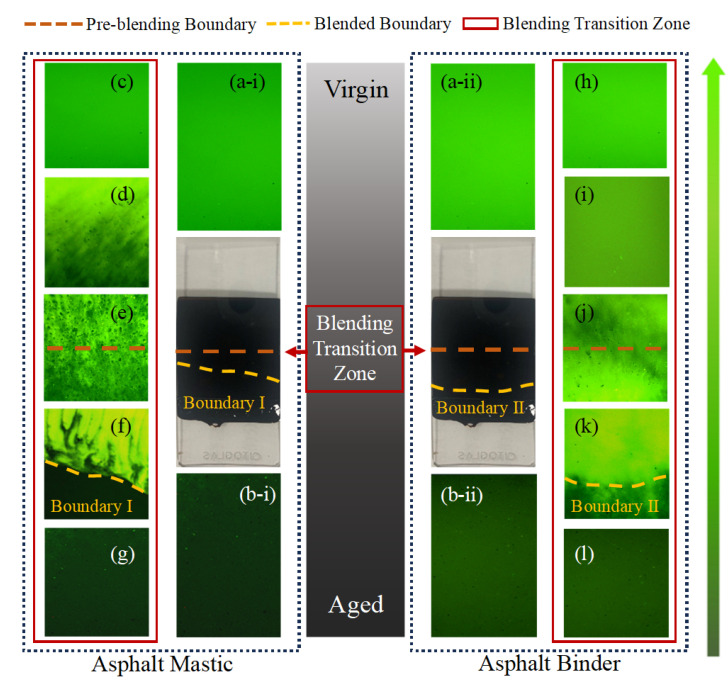
FM images of virgin aged asphalt binder and virgin aged asphalt mastic: (**a-i**,**a-ii**) virgin asphalt binder; (**b-i**,**b-ii**) aged asphalt binder or aged asphalt mastic; (**c**–**g**) blending transition zones of the virgin aged asphalt mastic; (**h**–**l**) blending transition zones of the virgin aged asphalt binder.

Furthermore, we further explored the blending behavior of virgin aged binder and mastic through FM. Figure 9 displays the fluorescence microscopy observations of virgin aged binder and mastic samples blended at 180 °C for 240 s. Each image is divided into three zones: the upper layer is virgin asphalt binder, the middle layer is the blending transition zone, and the lower layer represents aged asphalt binder or aged asphalt mastic. A comparison of Figure 9(a-i,a-ii,b-i,b-ii) reveals significant differences in fluorescence brightness and color. The aged materials exhibit darker fluorescence due to their lower content of aromatic and resin components, which are the main contributors to fluorescence. In contrast, the virgin asphalt binder regions are notably brighter, indicating a higher proportion of mobile maltenes. This pattern is consistent across both the asphalt and asphalt mastic systems. Notably, in Figure 9c–g, the regenerated asphalt or asphalt mastic shows intermediate brightness between the virgin and aged phases, suggesting partial diffusion and component exchange during the blending process. This can be explained by changes in SARA fractions during aging: as aromatics and resins degrade, fluorescence diminishes, making aged materials appear darker under FM [64]. Furthermore, oxidative aging leads to the formation of polar oxygen-containing functional groups such as carbonyl (C=O) and sulfoxide (S=O), which quench fluorescence by disrupting the conjugated aromatic structures. These transformations reduce the overall fluorescence emission intensity, directly affecting the brightness observed in the fluorescence microscopy images.

Moreover, as blending progresses, the virgin asphalt binder gradually permeates the aged layer, forming an interwoven structure within the blending transition zone. However, distinct boundaries and flaky residuals are still visible in the virgin aged asphalt mastic system, indicating incomplete integration. In contrast, as shown in Figure 9h–l, the virgin aged asphalt binder blend exhibits a more homogeneous interface with no obvious boundaries, suggesting a more thorough blending process. Figure 9f,k further highlight this distinction, showing that the interfacial boundary progressively shifts into the aged region, evidence of directional diffusion of virgin binder into the aged phase. Interestingly, the boundary in the asphalt mastic blend is positioned closer to the virgin binder than that in the asphalt blend, indicating a lower blending degree. This inferior blending performance may be attributed to the mineral fillers in aged asphalt mastic, which can form agglomerated structures that hinder virgin asphalt binder penetration and reduce molecular mobility, thereby impeding the diffusion and homogenization process. Moreover, the diffusion-limiting effect in asphalt mastic is also corroborated by the SEM observations. As shown in Figure 8, the interfacial boundary in the mastic system exhibits a pronounced gear-like or serrated morphology, contrasting with the smoother and more continuous transition zone observed in the asphalt binder system. This jagged interface further supports the inference that the presence of mineral fillers in asphalt mastic imposes significant resistance to the diffusion of virgin binder, resulting in a lower degree of blending. Additionally, these fillers increase viscosity and create physical barriers that further limit the DoB. However, regardless of whether fillers are present or not, fluorescence discontinuity always exists, reflecting that there is still a significant diffusion-limiting effect in both systems.

#### 3.3.2. Quantitative Evaluation of Blending Degree

**Figure 10:**
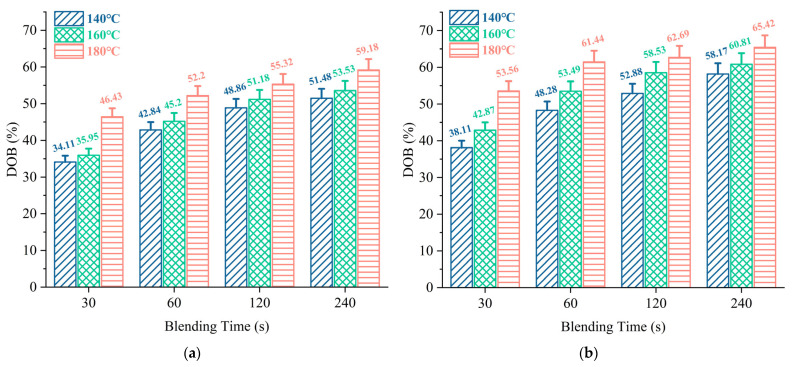
DOB values under different conditions: (**a**) asphalt mastic; (**b**) asphalt binder.

In addition, the DoB between virgin aged binder under varying blending conditions was quantified, and the results are illustrated in Figure 10. It can be observed from Figure 10a that the DoB values of virgin aged asphalt mastics generally increase with rising blending temperature and blending time, indicating that more aged asphalt mastic becomes mobilized and blends with the virgin binder. At a blending temperature of 180 °C, the DoB rises from 46.43% to 55.32% as the mixing duration extends from 30 s to 120 s. However, extending the mixing time to 240 s results in a marginal increase in DoB to 59.18%, suggesting that blending primarily occurs in the early mixing stage. It is attributed to the continuous molecular exchange at the interfacial layers between the virgin binder and aged mastic. During blending, the content of virgin binder in the diffusion phase gradually decreases, while its content in the receptor phase increases rapidly. The resultant weakening of the concentration gradient between the two phases leads to insufficient driving force for molecular diffusion across the fusion interface. Furthermore, it is evident that raising the blending temperature also effectively enhances the DoB between virgin aged mastic. With blending temperatures set to 140 °C and 160 °C, the DoB remains relatively low, not exceeding 55%. However, as the temperature rises to 180 °C, the DoB increases by nearly 10%. There are two reasons, as follows: (1) Brownian motion-driven diffusion: The blending and migration behavior of virgin aged mastic is governed by Brownian motion, which is fundamentally a thermal motion of molecules. Elevated temperatures intensify molecular movement, thereby strengthening the blending and diffusion processes. (2) Viscoelastic transition: As a viscoelastic material, asphalt exhibits high viscosity at lower temperatures, creating substantial resistance to blending. With temperature elevation, asphalt transitions into a fluid state, accompanied by a rapid reduction in viscosity. Concurrently, intermolecular forces weaken, enhancing the migration of virgin asphalt binder.

A comparison of Figure 10a,b reveals that virgin aged binder exhibits markedly greater blending efficiency compared to virgin aged asphalt mastic. Simultaneously, by observing the DoB values of (a) and (b), it is obvious that the disparity in DoB values between the two specimens is approximately 10% at blending temperatures of 140–160 °C, while, in terms of the blending time, the difference in the DoB values of the two samples is approximately 7%. This demonstrates that blending temperature plays a critical role in promoting blending effectiveness, as higher temperatures reduce viscosity and improve fluidity, thereby enhancing the degree of blending. However, the potential aging of asphalt at elevated temperatures must also be considered.

### 3.4. Correlation Between Mobilization and Blending

**Figure 11:**
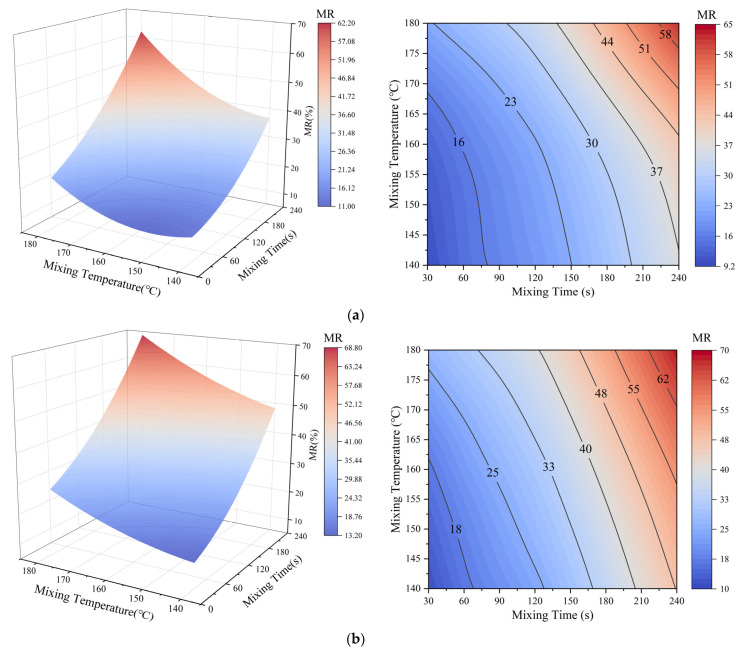
Correlation between MR and the blending temperature and time: (**a**) asphalt mastic; (**b**) asphalt binder.

To elucidate the multi-parameter coupling mechanism during the activation process of virgin aged binder and asphalt mastic, 3D surface fitting was conducted using blending time and temperature as variables. The resulting surface and contour plots of “mixing time-mixing temperature-MR” are shown in Figure 11. In Figure 11a, when the mixing time reaches 36 s at a temperature of 170 °C, the mobilization rate (MR) attains 18.54%, showing that the activation behavior of aged asphalt mastic under varying mixing conditions can be accurately characterized using the proposed approach. This provides valuable data and technical support for the visual evaluation and precise control of activation levels on the surface of RAP. Furthermore, a comparison with Figure 11b reveals that MR increases with both mixing temperature and time in both virgin aged binder and virgin aged asphalt mastic systems. This improvement is mainly due to the migration of virgin binder into the aged phase at the interface, which promotes the mobilization and activation of aged components. Nevertheless, the MR values in the asphalt binder system are consistently higher than those in the asphalt mastic system. This disparity mainly arises from the presence of mineral fillers in the mastic, which obstruct molecular diffusion and reduce interfacial contact, thereby limiting activation efficiency. These findings consistent with prior observations. Moreover, the contour plots in Figure 11 show that MR increases more significantly with rising mixing temperature than with extended mixing time. Specifically, in Figure 11a, MR rises sharply from 16% to 58% when the temperature increases from 140 °C to 180 °C, a change of over 40 percentage points, whereas increasing mixing time from 30 s to 240 s results in a smaller MR increase of approximately 25 percentage points. This demonstrates that mixing temperature has a major influence on enhancing MR by accelerating molecular interdiffusion at the interface. While prolonged mixing time also contributes to MR growth, its effect is comparatively less pronounced. Therefore, ensuring adequate mixing temperature is essential for achieving high activation efficiency in both asphalt binder and asphalt mastic systems.

With the fitted equations in Figure 11 given in Equation (6):

(6) $$Z=Z_{0}-a\times X-b\times Y+10^{-4}\times (c\times X^{2}+d\times Y^{2}+f\times X\times Y),$$

where *X* represents the mixing time (s), *Y* represents the mixing temperature (°C) and *Z* represents MR (%). The parameters and their corresponding values in Equation (6) are shown in Table 3.

In Equation (6), the linear terms *a* and *b* represent the primary sensitivities of the MR to changes in mixing time and mixing temperature, respectively. Specifically, the fitted coefficients presented in Table 3 indicate that the magnitude of *b* (–4.22) is more than ten-times that of *a* (–0.38), confirming that thermal activation is the primary driver of MR improvement in asphalt mastic. This trend is further illustrated by the contour and 3D surface plots in Figure 11, where the gradient of MR along the temperature axis is significantly steeper than along the time axis. The second-order coefficients *c* and *d* account for nonlinear behaviors such as plateau effects, reflecting the fact that MR does not increase indefinitely with prolonged mixing or elevated temperature due to physical limitations like interfacial saturation and filler obstruction. The interaction coefficient *f* represents the synergistic influence of time and temperature when varied simultaneously. A positive *f* suggests that a combination of moderate time and temperature may yield better mobilization than increasing one factor alone. Furthermore, the high coefficient of determination (*R*^2^ > 0.97) indicates excellent agreement between the predicted and experimental MR values, confirming the model’s robustness and suitability for predictive applications. This strong fit ensures that the model can reliably estimate mobilization behavior under various mixing conditions, thus providing a valuable tool for guiding RAP processing strategies and mixture design.

**Figure 12:**
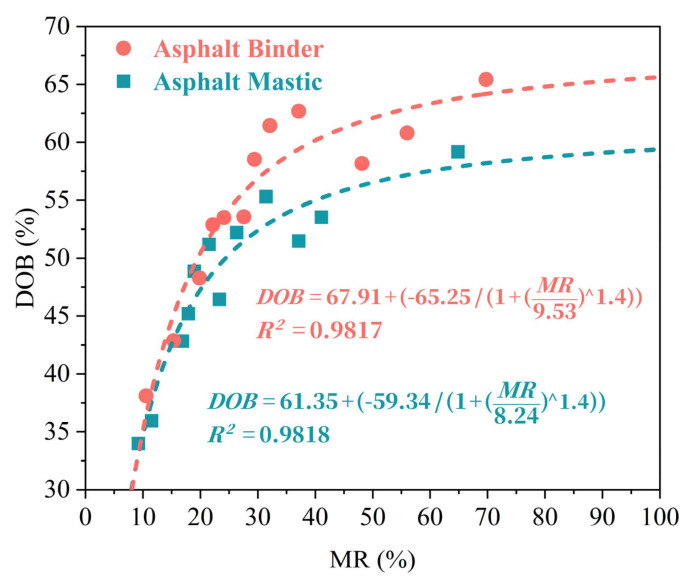
Correlation between *DOB* and *MR*.

To reveal the intrinsic coupling relationship and evolution trend between MR and DoB, a nonlinear fitting function was applied, as illustrated in Figure 12. The results show that DoB increases progressively with rising MR in both virgin aged asphalt binder and mastic systems. This trend indicates that the activation of aged components generates a significant concentration gradient at the interface, which promotes the diffusion and infiltration of light fractions from the virgin binder into the aged phase. Therefore, the enhancement of MR serves as a prerequisite for effective blending. Moreover, the relationship between MR and DoB is distinctly nonlinear. At the initial stage of blending, DoB exhibits a sharp increase in response to a relatively small rise in MR. Specifically, when MR increases from 10% to 40%, DoB rises significantly, indicating that limited molecular migration is sufficient to markedly enhance interfacial adhesion. However, when MR exceeds 40%, the growth rate of DoB begins to slow and gradually approaches a plateau. This deceleration is primarily attributed to interfacial saturation (indicating the extent to which the interface is occupied or saturated by diffused binder components, limiting further blending) and the development of structural stability, which impose both thermodynamic and physical constraints on further molecular diffusion. The nonlinear growth pattern highlights that, although mobilization facilitates blending, an increase in MR does not linearly translate into a proportional increase in DoB. More importantly, even when the MR approaches 100%, the DoB often remains incomplete. This indicates that mobilization alone is insufficient to achieve full interdiffusion. Without adequate mixing time or elevated temperature, the mobilized molecules may fail to fully penetrate and integrate into the aged asphalt matrix. Thus, maximizing blending efficiency requires not only a high MR but also favorable blending conditions. In practical engineering environments, even at 180 °C and 120 s, MR reached only 31.41% with a DoB of 55.32%, indicating a significant portion of aged binder remains inactive or poorly integrated. Overestimating effective binder content can lead to poor coating and premature pavement failure. Although increasing temperature and mixing time can improve blending, their effects plateau, and excessive heating may accelerate aging. Therefore, adjusting the virgin binder dosage and incorporating rejuvenators are essential to enhance interfacial diffusion, ensure adequate blending, and improve RAP mixture performance and durability.

Compared with previous studies, our approach further extends existing work by introducing the Composite Aging Index (CAI) to sensitively assess binder mobilization and using MGV-derived DoB alongside SEM-FM imaging to reveal interfacial limitations on blending. We establish a nonlinear MR–DoB relationship that connects molecular activation to macroscopic blending behavior. This integrated perspective advances the understanding of RAP regeneration mechanisms and supports more refined RAP mixture design.

## 4. Conclusions

Given the fundamental importance of activation and blending between virgin and aged asphalt mastic, this study quantitatively investigated their coupled mechanisms using a multiscale experimental approach integrating FTIR, SEM, FM, and digital image technique analysis. Moreover, these findings offer a scientific foundation for enhancing the performance of reclaimed asphalt mixtures and support sustainable pavement practices by increasing the efficient use of reclaimed materials, conserving natural resources, and minimizing environmental impact.

The main conclusions are as follows:

1. Regenerated asphalt mastic exhibited intermediate C=O and S=O peak intensities between virgin and aged samples, confirming partial dilution of oxidative groups. The absence of new peaks suggests a diffusion-dominated regeneration process. This supports the adoption of physical-compatibility-based strategies over chemical modification in practical recycling.
2. Although MR increased with higher temperatures (160–180 °C) and longer mixing times, it remained below 70%, indicating incomplete activation. Thus, time and temperature are necessary but insufficient alone, emphasizing the need for optimized conditions and material pairing to enhance recycling effectiveness.
3. A serrated, interlocked interface was observed between virgin and aged mastic, while binder interfaces showed smoother blending. This highlights the adverse role of mineral fillers in hindering interfacial diffusion, guiding the aggregate–mastic balance in mixture design.
4. Under FM, aged binders appeared darker due to aromatic loss, and blending boundaries shifted toward the aged side, indicating dominant virgin binder diffusion. The deeper penetration of binder over mastic reflects higher molecular mobility, informing targeted treatment of different asphalt phases in recycling.
5. Binder systems exhibited higher DoB than mastic, with temperature and time both promoting blending. Early-stage heating proved especially effective, suggesting that front-loaded thermal input may improve blending efficiency in industrial applications.
6. Mixing temperature influenced aged binder mobilization more than mixing time. Although DoB increased rapidly at 10–40% MR, it plateaued thereafter, showing that mobilization alone does not ensure full blending. Therefore, when higher temperature or longer mixing are impractical, adding rejuvenators is key to enhancing compatibility and diffusion between aged and virgin binders.

While this study focused on microscopic observations of binder blending behavior, future work will aim to correlate these findings with macroscopic mechanical performance metrics to establish more direct performance-based implications. In parallel, the proposed second-order polynomial model, developed based on controlled laboratory data, offers a useful foundation for interpreting blending behavior. To further enhance its applicability and reliability under broader engineering scenarios, including various material systems and field processing conditions, future studies are encouraged to incorporate independent datasets and explore potential refinements of the model. These efforts will help strengthen the linkage between microscale mechanisms and practical performance outcomes.

## Figures and Tables

**Table 1:** Technical indicators of virgin and aged asphalt binder.

Property	Test Result		Standard Specification
	Virgin Asphalt Binder	Aged Asphalt Binder	
Penetration (25 °C, 0.1 mm)	66.20	35.00	ASTM D5 [51]
Softening Point (°C)	48.55	68.00	ASTM D36 [52]
Ductility (15 °C, cm)	146.00	25.20	ASTM D113 [53]
Viscosity (135 °C, Pa∙s)	0.46	1.31	ASTM D4402 [54]

**Table 2:** Material composition of the regenerated asphalt mastic.

Virgin Aggregate	RAP	Virgin Binder	RAP Binder	Total Binder
45.5%	50.0%	1.6%	2.9%	4.5%

**Table 3:** Parameter values of asphalt mastic and asphalt binder.

Parameter	Value	
	Asphalt Mastic	Asphalt Binder
Z_0_	453	221
a	−0.38	−0.06
b	−4.22	−1.42
c	2.1	2.5
d	0.01	15
f	12	1
R^2^	0.9757	0.9839

## Data Availability

The data in this study were obtained through experiments. Some of the experimental procedures are openly available in [A blending efficiency model for virgin and aged binders in recycled asphalt mixtures based on blending temperature and duration] at [https://doi.org/10.1016/j.resconrec.2020.104957], reference number [57].
